# Chemical and Physical Characteristics of Doxorubicin Hydrochloride Drug-Doped Salmon DNA Thin Films

**DOI:** 10.1038/srep12722

**Published:** 2015-07-31

**Authors:** Bramaramba Gnapareddy, Sreekantha Reddy Dugasani, Taewoo Ha, Bjorn Paulson, Taehyun Hwang, Taesung Kim, Jae Hoon Kim, Kyunghwan Oh, Sung Ha Park

**Affiliations:** 1 Sungkyunkwan Advanced Institute of Nanotechnology (SAINT), Sungkyunkwan University, Suwon 440-746, Korea; 2 Department of Physics, Sungkyunkwan University, Suwon 440-746, Korea; 3 Department of Physics, Yonsei University, Seoul 120-749, Korea; 4 School of Mechanical Engineering, Sungkyunkwan University, Suwon 440-746, Korea

## Abstract

Double-stranded salmon DNA (SDNA) was doped with doxorubicin hydrochloride drug molecules (DOX) to determine the binding between DOX and SDNA, and DOX optimum doping concentration in SDNA. SDNA thin films were prepared with various concentrations of DOX by drop-casting on oxygen plasma treated glass and quartz substrates. Fourier transform infrared (FTIR) spectroscopy was employed to investigate the binding sites for DOX in SDNA, and electrical and photoluminescence (PL) analyses were used to determine the optimum doping concentration of DOX. The FTIR spectra showed that up to a concentration of 30 μM of DOX, there was a tendency for binding with a periodic orientation via intercalation between nucleosides. The current and PL intensity increased as the DOX concentration increased up to 30 μM, and then as the concentration of DOX further increased, we observed a decrease in current as well as PL quenching. Finally, the optical band gap and second band onset of the transmittance spectra were analyzed to further verify the DOX binding and optimum doping concentration into SDNA thin films as a function of the DOX concentration.

DNA molecules can be extracted from salmon fish by using a low-cost enzyme isolation process^1^,^2^. The resulting double-stranded salmon DNA (SDNA) is biodegradable, bio-absorbable, and non-toxic and has potential for use in a variety of physical and medical applications, including optoelectronic devices, drug delivery, or biomedical sensors. An important, preliminary step to investigate the interaction of DNA with functionalized biomolecules and nanomaterials, such as antitumor drugs, proteins, ions, and nanoparticles, is to first confine the DNA molecules on a solid surface. As a consequence, the interaction of small molecules with DNA has become an emerging field of research^3^,^4^,^5^,^6^,^7^,^8^,^9^. To utilize SDNA as a container for drug delivery, it is crucial to understand the selective binding mechanism between the chosen drug and SDNA. A number of representative bindings are possible for SDNA, including intercalation between base-pairs, steric preference in major and minor grooves, and electrostatic interaction around the phosphate backbones. In general, small molecules are placed in a groove binding due to geometrical compatibility with specific base sequences and are intercalated with nucleobases through hydrogen bonding and π-π stacking^10^. Doxorubicin hydrochloride (DOX) is a well-known anthraquinone anticancer drug molecule that is extensively used in anthracycline, and it has been proven effective against various human malignancies, including various types of cancers^11^,^12^,^13^,^14^.

In this study, we fabricated and characterized SDNA thin films doped with DOX in order to understand the binding phenomena between DOX and SDNA and to evaluate the optimum doping concentration (*C*_*o*_) of DOX. *C*_*o*_ is the DOX concentration ([DOX]) in SDNA that provides the maximum current and highest photoluminescence (PL) intensity without any precipitation, which are desirable characteristics for biomedical and nanotechnological applications. The SDNA thin films were doped with various [DOX] and were subsequently drop-casted on oxygen plasma treated glass and quartz substrates. Treatment with oxygen plasma changes the behavior of the surfaces of the substrates from hydrophobic to hydrophilic, and so can be helpful in forming uniform thin films. The thin films were fabricated and were subsequently characterized through several methods. Fourier transform infrared (FTIR) spectroscopy was used to understand the DOX bindings, current–voltage (*I−V*) curves were obtained through a semiconductor parameter analyzer to estimate the *C*_*o*_ of DOX, and the PL spectra were captured using a fluorimeter to evaluate both the quenching mechanism and the *C*_*o*_ of DOX in SDNA. In addition, we employed ultraviolet-visible (UV-Vis) spectrophotometry to investigate the energy band gap and optical transition in order to further confirm the DOX bindings and the *C*_*o*_ of the SDNA thin films.

## Results

**Figure 1:**
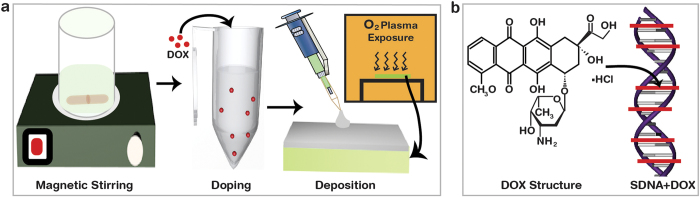
Schematic diagrams of SDNA thin film preparation, molecular structure of DOX, and DOX coordination in SDNA. (**a**) Preparation of SDNA sample by magnetic stirring, DOX doping into the SDNA sample, substrate cleaning with O_2_ plasma, and fabrication of DOX-doped SDNA thin film via drop-casting. (**b**) Molecular structure of DOX, and representative scheme of the DOX coordination into SDNA. (All schematic diagrams in this figure were drawn by B. Gnapareddy.)

The preparation process for DOX-doped SDNA thin films, the molecular structure of DOX, and the DOX coordination sites in SDNA were shown in Fig. 1. To prepare the SDNA solution, 0.1 g of SDNA were dissolved in de-ionized (DI) water, and the solution was magnetically stirred to achieve a homogeneous mixture of SDNA in solution (see the Methods section for a full description of the SDNA sample preparation). 200 μL of the final SDNA solution at a concentration of 0.5 wt. % were placed into a test tube, and various amounts of DOX were added (0, 10, 20, 30, 40, and 50 μM). The substrates used in this study were treated with oxygen plasma to provide better adhesion, and 15 μL of the DOX-doped SDNA solution were then drop-casted on the substrates to form flat thin films with thicknesses of ~100 nm.

**Figure 2:**
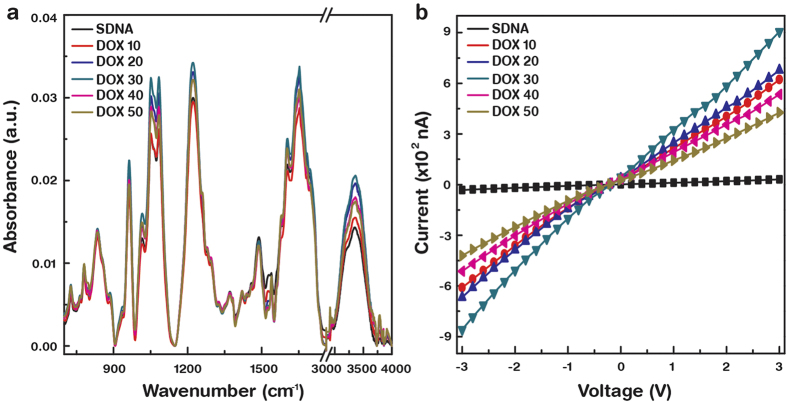
Fourier transform infrared spectra and current−voltage characteristics with various concentrations of DOX in SDNA thin films. (**a**) FTIR absorption spectra of DOX-doped SDNA thin films in the wavelength range from 4000 to 700 cm^−1^. The colour lines indicate SDNA absorption spectra with various concentrations (10, 20, 30, 40, and 50 μM) of DOX as well as pristine SDNA. (**b**) Variation of current-voltage characteristics of DOX-doped SDNA thin films as a function of DOX concentration.

FTIR spectroscopy was employed in the spectral range from 4000 to 700 cm^−1^ to investigate the binding mechanism for DOX into specific chemical groups of the SDNA molecules. Figure 2a shows the FTIR−attenuated total reflection spectra for SDNA thin films doped with various [DOX] after subtracting the background glass spectrum. Absorption peaks at 3360, 1700, 1653, 1603, 1488, 1420 1373, 1220, 1082, 1050, 1015, 963, 938, 835, and 780 cm^−1^ indicate standard *B*−form DNA characteristics. Table 1 presents the FTIR spectrum of the SDNA in terms of the chemical bonds/functional groups and their peak position assignments^15^,^16^,^17^. The absorption bands were divided into three regions: 3600 − 3000 cm^−1^ for water OH stretching, 1800 − 1300 cm^−1^ for nucleobases, and 1250 − 700 cm^−1^ for the sugar and phosphate backbone groups. For the nucleobases, the absorption peaks at 1700 cm^−1^ show the chemical features of guanine C = O stretching, the peaks at 1653 cm^−1^ show thymine C2 = O stretching, the peaks at 1603 cm^−1^ show adenine C7 = N stretching, and the peaks at 1488 cm^−1^ for the cytosine in-plane vibration. The other absorption peaks at 1420 and 1373 cm^−1^ indicate cytosine and guanine. The absorption peaks at 1220 and 1082 cm^−1^ are the anti-symmetric and symmetric stretching vibration of the phosphate groups, respectively. The absorption peak at 1050 cm^−1^ is typically assigned to C−O deoxyribose, the peak at 1014 cm^−1^ to P−O or C−O stretching, the peak at 963 cm^−1^ to the C−C and C−O of deoxyribose skeletal motions, the peak at 938 cm^−1^ to adenine-thymine base pairs, the peak at 835 cm^−1^ to deoxyribose-phosphate, and the peak at 780 cm^−1^ to sugar phosphate vibration modes.

The *I−V* characteristics of DOX-doped SDNA thin films as a function of [DOX] were discussed in Fig. 2b. Silver electrodes with a fixed channel width of ~1 mm were used for all samples. The measurements were conducted using a two terminal probe station at ambient temperature and pressure. *I* initially increased as [DOX] increased up to *C*_*o*_ and then decreased as [DOX] increased further. The experimental data indicate that 30 μM of DOX in SDNA (*C*_*o*_) provided a nine-fold increase in *I* relative to SDNA without DOX. We noticed slightly nonlinear *I−V* characteristics in the curves that were measured, which might be the result of an asymmetric contact potential between the silver electrodes and the SDNA thin films. The contact mechanism between the metals and the DNA molecules relied on the work function of the metal electrodes, which will be discussed elsewhere.

**Figure 3:**
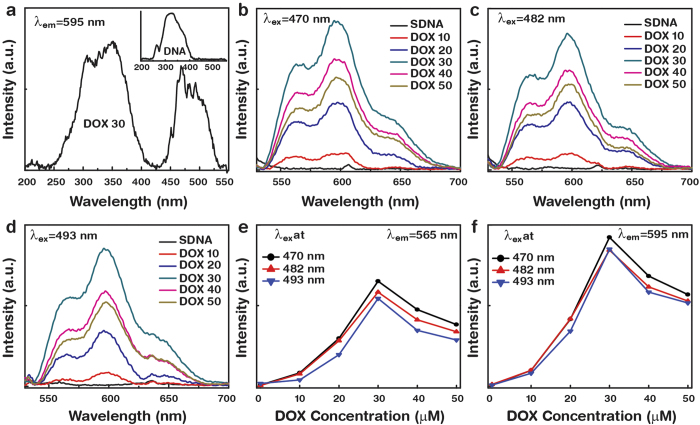
Photoluminescence excitation spectrum, photoluminescence, and its analysis of DOX-doped SDNA thin films at various excitation wavelengths. (**a**) The photoluminescence excitation spectra for 30 μM of DOX-doped SDNA and for pristine SDNA (inset). Photoluminescence spectra of DOX-doped SDNA thin films at distinct excitation wavelengths, (**b**) λ_ex_ = 470 nm, (**c**) λ_ex_ = 482 nm, and (**d**) λ_ex_ = 493 nm. Analysis of the photoluminescence intensity at (**e**) 565 nm and (**f**) 595 nm as a function of DOX concentration.

Figure 3 presented the photoluminescence excitation and PL spectra of the DOX-doped SDNA thin films at various excitation wavelengths (λ_ex_). The excitation spectra of pristine SDNA and of the SDNA thin films doped with DOX at *C*_*o*_ were monitored at a fixed emission wavelength (λ_em_) of 595 nm. Figure 3a shows the excitation spectra of 30 μM of DOX-doped SDNA and pristine SDNA thin films. Pristine SDNA shows characteristic excitation peaks at 264 and 320 nm (inset in Fig. 3a) while DOX-doped SDNA thin films reveal appreciable characteristic peaks of DOX molecules at 470, 482, and 493 nm in addition to those exhibited by pristine SDNA. Figs 3b–d show the PL spectra of the DOX-doped SDNA thin films as a function of [DOX] at various λ_ex_. These spectra present two characteristic emission peaks of DOX at 565 and 595 nm that could not be observed from the pristine SDNA sample, and we also noticed a change in the intensity as a function of [DOX], which revealed the degree of the chemical bindings of DOX in SDNA molecules. The emission intensities increased as [DOX] increased up to *C*_*o*_, and then decreased as [DOX] increased further, as shown in Fig. 3e,f. We found that the PL emission at a particular λ_ex_ of 470 nm was the strongest relative to other λ_ex_, which means that an λ_ex_ of 470 nm is more appropriate for energy transfer between the DOX and SDNA molecules.

**Figure 4:**
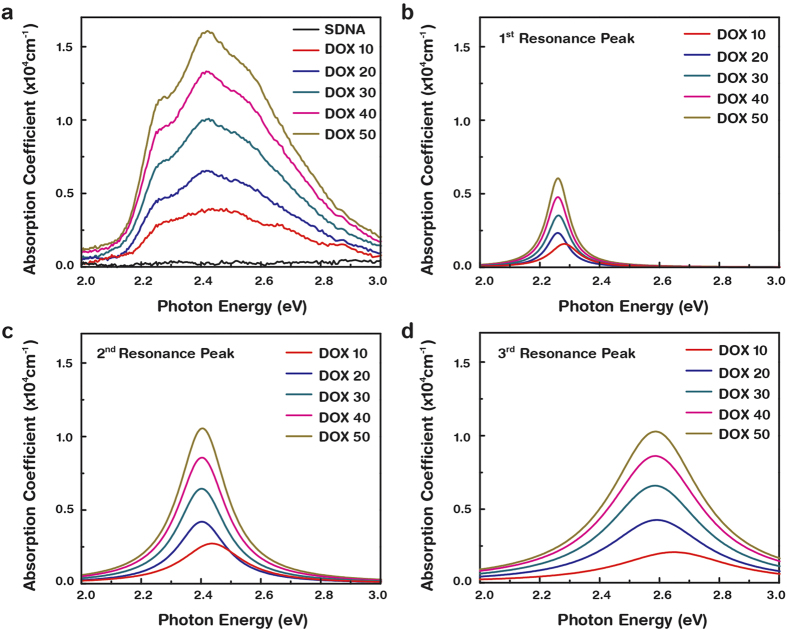
Variation of absorption coefficient as a function of photon energy for DOX-doped SDNA thin films. (**a**) Total absorption spectrum, (**b**) 1^st^, (**c**) 2^nd^, and (**d**) 3^rd^ resonance peaks as a function of photon energy extracted from total absorption spectrum using Lorentzian function.

UV−Vis spectrophotometry was conducted at ambient temperature and atmospheric pressure, and we determined the optical band gap as well as the second band onset of the DOX-doped SDNA. Figure 4a shows the absorption coefficient (*α*) with a variation in the photon energy (*E*) of the DOX-doped SDNA at a fixed [DOX] of 0, 10, 20, 30, 40, and 50 μM. Here the three resonance *α* peaks observed at specific *E* of 2.26, 2.42, and 2.55 eV, as shown in Fig. 4b–d, were the intrinsic DOX absorption characteristics^18^. The resonance *α* peaks were extracted from a general single resonance state obtained from Fig. 4a by using the Lorentzian function to independently visualize each resonance *α* peak. All of the resonance *α* peaks were obtained after removing the background signal generated by the quartz substrate. Three resonance *α* spectra show a red-shift in peak position between a [DOX] of 10 and 20 μM, which correspond to an *E* from 2.44 to 2.40 eV. This red-shift is a result of the increasing [DOX] in SDNA, which is related to the absorption feature of pristine DOX at ~2.51 eV^19^. Above 20 μM of [DOX], shifts in the peak positions could be hardly observed because a [DOX] of around 20–30 μM in SDNA is optimally placed at the designated sites in SDNA.

**Figure 5:**
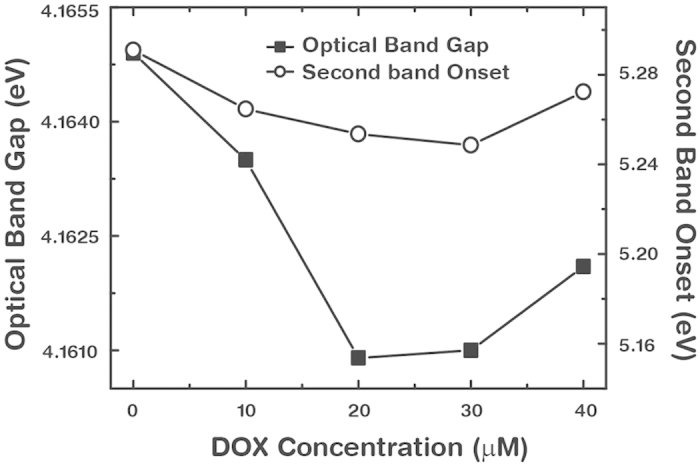
Optical band gap and second band onset of DOX-doped SDNA thin films. Variation of the optical band gaps and second band onsets with various concentrations of DOX.

The optical transmittance spectra were analyzed through linear fitting to determine the band gaps of the DOX-doped SDNA as a function of [DOX]. The optical band gap can be extracted from the lower energy side of the first absorption band because the DOX-doped SDNA thin films have a sufficient thickness of ~100 nm, and a second band onset was determined through an analysis of the super-continuum current-carrying states after making intra-band transitions to the bottom of the conduction. As a result of the proper thickness of the thin film, our analysis methodology for the first band absorption energy provided greater precision than the highest occupied molecular orbital (HOMO)—lowest unoccupied molecular orbital (LUMO) band analysis that is related with the electronic transition of π → π*, essentially a set of superimposed HOMO-LUMO transition of the corresponding character in a free DNA molecule. The first absorption band revealed as a continuum state due to superposition of many single π → π* energy states, and the states occurred due to an interaction between DOX and SDNA. The optical band gaps of the DOX-doped SDNA thin films with various [DOX] varied from 4.164 to 4.161 eV, as shown in Fig. 5. The slight difference of ~10% in the band gap energies between the SDNA thin film discussed here and that of DNA molecules that were previously reported might be due to the differences in buffer conditions, thickness, length, and amount of the DNA used as well as interfacial characteristics^20^,^21^,^22^,^23^. In addition, we presented the onset energy of the second optical absorption band, which evolved with DOX in a manner similar as that of the optical band gap (Fig. 5). The behavior of the second band onset was similar to that of the optical band gap, with optimum conditions at 30 μM of [DOX] into SDNA.

## Discussion

The FTIR revealed DOX binding configuration into SDNA with various [DOX] and offered evidence of a saturation dopant [DOX] directly related to *C*_*o*_. The FTIR spectra revealed that DOX with 30 μM ( = *C*_*o*_) in SDNA had the highest absorbance intensities, which might be related to binding with periodic orientation via an intercalation mode between the nucleosides. Due to the shape (roughly plane) and size (slightly larger than single nucleotide), the DOXs were placed between layers of base-pairs of SDNA duplexes through chemical bonds with nucleosides, as shown in Fig. 1b. An appreciable enhancement in the absorption band intensities of the nucleosides and phosphate backbones of SDNA was evident up to *C*_*o*_, but a further increase of [DOX] in SDNA resulted in a decrease in the absorption band intensities, possibly as a result of the random orientation, electrostatic and nonspecific bindings of DOX to SDNA. Therefore, a higher [DOX] relative to *C*_*o*_ might produce undesired stress and strain on helical DNA molecules which cause a reduction in intensities. Although structural changes of SDNA in the presence of excess amount of DOX were hardly measureable, structural deformation due to stress and strain caused by a higher [DOX] than *C*_*o*_ was observed in the artificially designed DNA nanostructures (data were not shown). Zheng *et al.* reported absorption bands of pure DOX at 1088, 1285 and 1621 cm^−1^ to be the bending vibrations of the amino group; 1434 and 1586 cm^−1^ to the in-plane stretching vibrations of ring C–C; 1385 cm^−1^ to the phenyl ring vibration; and 3448 cm^−1^ to the broad band of O–H vibration^24^. Although the FTIR spectra of the DOX-doped SDNA thin films showed the similar features as pristine SDNA, an appreciable band intensity variation and a slight shift in the band position were observed as [DOX] varied.

The *I−V* characteristics of the DOX-doped SDNA thin films were measured as a function of [DOX] to observe the electrical behavior and to estimate *C*_*o*_. The data indicate that *I* increased at a [DOX] up to 30 μM and then decreased as [DOX] further increased, revealing an inverted V shape. This behavior suggests that the SDNA thin films with a 30 μM DOX could be chemically-bound between the nucleosides and physically-bound with phosphate groups with a maximum tolerance in the physical stress and strain in the DNA molecules. During the sweep the voltage, the DOX-doped SDNA showed a higher *I* that was a maximum of ~10 times larger than SDNA without DOX. This observation may be a result of electron hopping between the DOX and SDNA with shorter hopping distances and screened net charges of DOX that could effectively influence electron transport through the SDNA thin films. Above 30 μM of [DOX], the DOX molecules would nonspecifically bind with random orientations, causing a decrease in *I*. As a consequence of the bowing behavior of *I* as a function of [DOX], we assigned a [DOX] with a maximum *I* as *C*_*o*_ and anticipated the SDNA thin film with a DOX of *C*_*o*_ to be optimally aligned in terms of the conduction.

The PL can be used to explain the energy transfer mechanism between the DOX and SDNA in the thin films and can also provide an indirect clue of the existence of *C*_*o*_. The PL emerges from the release of a photon upon relaxation of the electron from the triplet excited state. Since the absorption band of DNA molecules is in the range between 250 to 280 nm, energy transfer occurred through internal conversion within the excited singlet state, then from the excited singlet state to the triplet state via intersystem crossing, and then to the emissive states^25^. The enhancement and quenching of the PL as [DOX] varied was indicative of the degree to which the DOX binding to SDNA occurred in designated and improper regions, respectively due to the size and shape of the DOX molecules. Although the yield and efficiency might be different due to the spectral broadness and change in intensity, the λ_em_ of all DOX-doped SDNA thin films were almost similar with respect to various λ_ex_ and [DOX]. In general, the fluorescence quenching of DOX in the presence of DNA was caused by doping of the drug molecules into the DNA^26^.

The variation in the optical band gaps and the second band onsets with various [DOX] were analyzed in order to understand the optical transition through DOX-doped SDNA thin films. A significant deviation (decrease) in the optical band gap occurred between 10 and 20 μM of DOX into SDNA. This result was consistent with the absorption spectra of DOX-doped SDNA shown in Fig. 4. With [DOX] greater than 20 μM, the optical band gaps gradually increased due to the DOX excess. The initial decrease in the optical band gap up to a certain [DOX] was in agreement with the situation in which the DOX coordinated with the SDNA. Beyond *C*_*o*_, DOX overfilling begins to introduce random orientations, as previously mentioned, causing a blue-shift in the optical band gap. The critical doping [DOX] for the optical band gap and for second band onset were determined to be 20 and 30 μM, respectively, which were pretty close to *C*_*o*_. Such trends in the energy band gaps and in the onset were consistent and were in good agreement with the FTIR absorption bands, *I−V* characteristics, and PL spectra that had been previously discussed. The optical band gap was supposed to represent the minimum energy to form an exciton, an electron-hole pair, even though this resulting two-body state rarely contributed to the transport process. In contrast, the second band onset energy state could form current-carrying states after making intra-band transitions that occur frequently in pentacene-based organic devices^27^,^28^.

In conclusion, we have successfully fabricated SDNA thin films doped with DOX drug molecules on the given substrate via drop-casting. The concentration of the dopant [DOX] was controlled, and we analyzed the preferred coordination binding sites and the optimum doping concentration of DOX into SDNA via FTIR, *I-V*, PL and band gap measurements. The FTIR spectra revealed a chemical binding preference for DOX with periodic orientation between nucleosides up to the optimum doping concentration *C*_*o*_ (30 μM). The *I* and PL measurements showed an increase as [DOX] increased until *C*_*o*_, and then a decrease in *I* and a quenching of PL were observed as [DOX] increased further. Further tests to confirm the DOX bindings and *C*_*o*_ were carried out by analyzing the optical band gap and second band onset using the optical transmittance spectra. DNA with functionalized materials has diverse applications in bionanotechnology. For example, DNA can be used either as a container for specific drugs in biomedical applications or as a template with functionalized nanomaterials for bio-nano devices. It is crucial to find the appropriate optimum ratio of functionalized materials and DNA and to understand the binding phenomenon between them in order to construct efficient devices and sensors made with DNA that offer better performance.

## Methods

### SDNA sample solution preparation

To prepare the SDNA solution (Chitose Institute of Science and Technology, Hokkaido, Japan), 0.1 g of SDNA were dissolved in 10 mL of DI water (the resulting solution has a concentration of 1 wt. % SDNA in DI water), followed by magnetic stirring (about 1000 rpm for 10 hours at room temperature) to achieve a homogeneous mixture of SDNA in solvent and to remove air bubbles in the SDNA solution. After preparing the 1 wt. % SDNA sample solution, we diluted it into a final concentration of 0.5 wt. % SDNA solution.

### Doxorubicin drug doping into SDNA

The SDNA sample solution with 0.5 wt. % was pippetted into new Eppendorf-tubes with a total volume of 200 μL in each. We added an appropriate amount of DOX, *i.e.*, 0, 10, 20, 30, 40, and 50 μM, into each SDNA sample solution followed by vortex mixing for 5 minutes and incubation for 24 hours at room temperature in order to obtain a homogeneous mixture of DOX-doped SDNA samples.

### Thin film fabrication on the given substrates

Glass (for FTIR, and *I-V*) and quartz (for PL, and band gap) substrates with size of 5 × 5 mm^2^ were treated with O_2_ plasma for surface modification. The O_2_ plasma treatment introduced the silanol group into the substrate, and this changed the charge from neutral to negative resulting in a change from hydrophobic to hydrophilic. An O_2_ plasma cleaner (Plasma Processing System, CUTE−1 MP/R, Gyeonggi, Korea) was used in this study. The O_2_ plasma process was performed under the following conditions: 50 W power, 5 × 10^−2^ Torr base pressure, 47 SCCM oxygen flow rate, 7.8 × 10^−1^ Torr working oxygen pressure, and plasma generation time of 5 minutes for glass and 10 minutes for quartz substrates. After the O_2_ plasma exposure, 15 μL of DOX-doped SDNA sample solution were used to fabricate the thin films via drop-casting.

### Fourier transform infrared spectroscopy measurement

The FTIR spectra of the DOX-doped SDNA thin films in the range from 4000 to 700 cm^−1^ were recorded by a TENSOR 27 spectrometer [Detector: MIR_ATR (ZnSe), Bruker Inc., USA]. 32 scans were co-added and averaged with a resolution of 4 cm^−1^. The data in the FTIR spectra used in the present study were analyzed by subtracting the background spectrum produced by bare glass.

### Current-voltage measurement

The electrical properties of the DOX-doped SDNA thin films on glass were determined by using a semiconductor parameter analyzer (4200−SCS, Keithley Instruments Inc., USA). A silver paste was deposited on the DOX-doped SDNA thin film to form metal electrodes with a channel gap length of approximately 1 mm.

### Photoluminescence measurement

A Photon Technology International Fluorimeter (USA) with a Xe-arc lamp power of 60 W was used to obtain photoluminescence and excitation spectra of the DOX−doped DNA thin films on quartz at room temperature. The excitation spectra were obtained at a fixed emission wavelength, and the emission spectra were measured by exciting the samples at three different wavelengths of 470, 482, and 493 nm.

### Optical band gap measurement

A Varian Cary 5G spectrophotometer was used to conduct the optical transmission measurements of the DOX−doped DNA thin films on quartz in the near-infrared, visible, and ultraviolet regions (wavelengths between 1200 and 190 nm). The spectrophotometer was equipped with two light sources: a deuterium arc lamp (near-infrared and visible) and a quartz W−halogen lamp (ultraviolet). It also has two detectors: a cooled PbS detector for the near-infrared region and a photomultiplier tube for the visible and ultraviolet regions. The spectrophotometer measured the frequency-dependent light intensity passing either through a vacuum or through the sample.

## Additional Information

**How to cite this article**: Gnapareddy, B. *et al.* Chemical and Physical Characteristics of Doxorubicin Hydrochloride Drug-Doped Salmon DNA Thin Films. *Sci. Rep.* **5**, 12722; doi: 10.1038/srep12722 (2015).

## Figures and Tables

**Table 1:** Fourier transform infrared spectrum absorption band positions and corresponding band assignments of the SDNA thin film.

**No**	**Band Position (cm^−1^)**	**Band Assignments**
1	780	Sugar phosphate vibration
2	835	Deoxyribose phosphate
3	938	Adenine−Thymine base pairs
4	963	C−C and C−O of deoxyribose skeletal motion
5	1014	P−O or C−O stretching
6	1050	C−O deoxyribose stretching
7	1083	Phosphate symmetric stretching
8	1221	Phosphate antisymmetric stretching
9	1372	Cytosine and Guanine
10	1420	Cytosine and Guanine
11	1488	Cytosine (in-plane vibration)
12	1603	Adenine (C7=N stretching)
13	1653	Thymine (C2=O stretching)
14	1700	Guanine (C=O stretching)
15	3357	OH stretching
